# Audit of follow-up within 7 days on discharge from the mental health unit, Forth Valley Royal Hospital

**DOI:** 10.1192/bjo.2021.602

**Published:** 2021-06-18

**Authors:** Matthew Turner, Shaun Love, Fergus Douds, Anyssa Zebda

**Affiliations:** 1 Forth Valley Royal Hospital; 2 NHS Greater Glasgow and Clyde

## Abstract

**Aims:**

To determine compliance with the new discharge policy of review within 7-days for all General Adult Psychiatry patients discharged from Forth Valley Royal Hospital.

**Background:**

It is well established that there is an increased risk of suicide following discharge from Inpatient Psychiatric Wards. This risk is significantly increased in the first month, and particularly high in the first week.

In their 2016 Guidance, NICE recommends follow-up within 7 days of discharge. It is not known whether seven day follow-up reduces suicide risk but it is clearly an opportunity for risk assessment and management during a particularly risky period.

This standard was adopted by the General Adult Wards in Mental Health Unit at Forth Valley Royal Hospital in April 2019.

**Method:**

All discharges from Wards 1, 2 and 3, Forth Valley Royal Hospital were reviewed during three distinct, month-long periods:

November 2018 (prior to the introduction of the new discharge policy)

May 2019 (shortly after the introduction of the new discharge policy)

September 2019 (six months after the introduction of the new discharge policy)

A list was obtained from Medical Records of all General Adult patients discharged in these periods. The paper and electronic records were checked for each patient, and the first scheduled care episode post discharge was taken as follow-up.

**Result:**

In the1st round of audit (November 2018): 41 patients were discharged and 26 patients (64%) received follow-up within 7 days.

In the 2nd round of audit (May 2019): 46 patients were discharged, 39 patients (84%) received follow-up within 7 days.

In the 3rd round of the audit (September 2019), 50 patients were discharged and 49 (98%) received follow-up within 7 days.

**Conclusion:**

There has been a clear improvement in the provision of follow-up on discharge from the General Adult Psychiatry Wards in Forth Valley Royal Hospital.

The new discharge policy was implemented in April 2019 and a “Discharge Pause” was introduced (initially a sticker, now an electronic form) to be completed by the medical team at the point when it was decided to discharge.

Community Mental Health Teams have also been reminded of their need to facilitate seven day follow-up as a priority. A flowchart was produced in May 2019, which provided guidance as to who should provide the seven day follow-up.

